# 

**Published:** 1886-07

**Authors:** 


PAGE.

ORIGINAL COMMUNICATIONS:

Some Observations of the Uterine Sound. By W. W. Potter, M. D., . . . . . . . 543
Intubation of the Larynx. By A. R. Davidson, M. D., . . . . . . . . . . . 553
The Early Diagnosis of Pregnancy. By E. S. McKee, M. D., . . . . . . . 563

SOCIETY REPORTS:

Nineteenth Annual Meeting of the Central New York Medical Society, . . . . . 567
Buffalo Medical and Surgical Association, . 575

SELECTIONS:

Suture of Nerves, . . . . . . . . . . 578
The Etiology of Dysentery, . . . . . . 579
The Treatment of Sterility, . . . . . . 580

MEDICAL NEWS:

Vicarious Menstruation, . . . . . . . 581
Chancre, . . . . . . . . . . . . . . 581

EDITORIAL:

Urethane, . . . . . . . . . . . . 582
College Faculties, . . . . . . . . . . 583
Dispensaries, . . . . . . . . . . . . 585

REVIEWS:

International Encyclopœdia of Surgery. By John Ashurst, Jr., M. D. . . . . . 586
Diseases of the Digestive Organs in Infancy and Childhood. By Louis Starr, M. D., 587
Diseases of the Spinal Cord. By Byron Bramwell, M. D., . . . . . . . . . 588
Practical Clinical Lessons on Syphilis and the Genito-Urinary Diseases. By Fessenden N. Otis, M. D., . . . . . . 588

### Index to Advertisements of Half a Page or Over.

PAGE.

